# Uneven Implications of Lockdown Amid COVID-19 in India: From Harassment, Stigma, Crime, and Internally Displaced People to Stress and Coping Strategies in the Middle/Upper Class

**DOI:** 10.3390/bs12100348

**Published:** 2022-09-21

**Authors:** Shagufta Nasir, Mohammad Ghazi Shahnawaz, Lydia Giménez-Llort

**Affiliations:** 1 Department of Psychiatry and Forensic Medicine, School of Medicine, Universitat Autònoma de Barcelona, 08193 Barcelona, Spain; 2 Department of Psychology, Jamia Millia Islamia, New Delhi 110025, India; 3 Institut de Neurociències, Universitat Autònoma de Barcelona, 08193 Barcelona, Spain

**Keywords:** national lockdown, COVID-19, content analysis, psychological and socioeconomic implications, vulnerability

## Abstract

A content analysis of an English Newspaper, The Times of India (the world’s largest newspaper by circulation) during the first national lockdown amid the COVID-19 pandemic identified nine different categories culled out from a total of 129 news categories reporting unprecedented COVID-19 stories. Half of them portrayed two sides of a coin: from daily wagers and migrant workers, including internally displaced people (23/129), harassment and stigma (4/129), and crime (3/129) to stressors and coping strategies for middle/upper class individuals (39/129). Reports evidenced increased vulnerability in the lower layers of Indian stratified society. Yet, two years later, the uneven implications on physical and mental health are scarcely studied by scientific researchers.

## 1. Introduction

The COVID-19 pandemic has created an unprecedented upheaval in the world. Within a short course of time, COVID-19 was declared a global pandemic, affecting over 213 countries and territories on six continents [1]. The COVID-19 pandemic has created far-reaching economic, physical, social, and mental health consequences all over the world [2]. According to the International Monetary Fund (IMF), the slowdown in the economy due to COVID-19 is the steepest since the Great Depression [3], and it is expected that full recovery will take a long time [4]. No one was excluded from the COVID-19 outbreak, it affected all sections of society, and was particularly detrimental to those social groups in vulnerable situations [5]. Many countries resorted to lockdowns to limit human contact and to reduce the spread of illness [6]. There is evidence that lockdown has affected people’s physical health [7], mental health [8], and social health [9]. However, the health and well-being of the people would be affected more in low- and middle-income countries than in developed nations [10]. In India, healthcare inequalities persist and are observed by disadvantaged members of society based on socioeconomic status, gender, and geographical location [11]. The burden of the COVID-19 pandemic has deepened inequality in LMIC countries more than ever before [12].

The purpose of the current study was to examine how the newspaper recognized and informed the impact of the sudden national lockdown on all sections of the Indian society amid the COVID-19 pandemic, as India was among one the countries that imposed a national lockdown from 25th March 2020 to 31st May 2020. In India, the national lockdown was extended over four phases, and each phase was marked by increasing relaxation. Reporting during novel public health emergencies with scientific knowledge is very challenging [13]. In this respect, traditional newspaper coverage is still the most reliable and valued public information source [14].

Newspaper reports have been used as a data source in the current research. Newspapers reflect the social and cultural values of a particular place and time, and often contain exclusive information with immediacy, which cannot be found anywhere else [15]. The newspaper mirrors the linguistic structure of a particular period. They are a substantial source of data for research in the arts, social sciences, and humanities [16]. Thus, the newspaper can be considered an important source of multifaceted information on a diverse range of issues impacting any society (Indian society, the locale of the present analysis) in the aftermath of the national lockdown during the COVID-19 pandemic.

The primary aim of the present study was to understand the impact of the sudden lockdown on the general population of India using newspaper content analysis. Our second aim was to examine the progression and translation of the identified categories into scientific research over a span of two years. For this purpose, two years later, which is considered a typical time period for scientific studies to result in reports, a second study was performed to screen the representation of the different categories on scientific databases.

## 2. Materials and Methods

### 2.1. Study Design and Data

The present study was developed in two stages. In the first stage, the newspaper reports using keywords “COVID-19”, “COVID”, and “pandemic” were scrutinized from the 25 March 2020, immediately after the announcement of the national lockdown, to the 7 April 2020, following up to two weeks. The Times of India (henceforth, TOI, New Delhi, India Edition) was chosen for the analysis as it is the oldest English- language daily newspaper in India, often hailed as the world’s largest newspaper by circulation [17]. The website of an online newspaper (e-paper) was used to identify the news articles published on the issues related to the COVID-19 pandemic. Since the translation of novel questions and individual/social needs into scientific outputs was not immediate, the second stage of the study was carried out after two years. Subsequently, according to the different thematic findings, an extensive scoping review of the literature was conducted to monitor the literature gap, investigate the conduct of the research, and identify the solutions to the problems tackled by the general public.

### 2.2. Content Analysis

We used content analysis to examine newspaper reports. Content analysis is a research technique for formulating replicable and suitable conclusions from data. It aims at offering knowledge, new insights, an illustration of facts, and a realistic guide to action [18]. It is a flexible technique as it allows the researcher to obtain the systematic interpretation of the textual, visual, or audible matter, such as newspaper editorials, television, news, advertisements, public speeches, and other verbal or non-verbal units of analysis [19]. Content analysis of newspapers is a valuable method to assess community opinions, advocacy, and change [20]. 

For the present study, the conventional approach to content analysis was used. Initially, we highlighted the exact words from the press reports representative of similar concepts. Next, the labels for codes were provided based on the reflection of the key thoughts. Then, codes were sorted into categories based on their similarities and differences from each other. Lastly, these emergent categories were organized for the meaningful interpretation of the news reports [21]. The purpose of this report was to assess the immediate impact of the sudden lockdown on the general population of India. With the help of the content analysis, a total of nine (*n* = 9) categories were identified in the study.

### 2.3. Scoping Review

The literature scoping review was performed following the guidelines of the PRISMA method for its correct elaboration. The framework for scoping review was: (1) identification of the objective of the review, (2) finding the relevant studies, (3) selecting studies to be included in the present study, (4) data extraction from the included studies, (5) summarizing and reporting the results.

Scientific publications from 2020–2022 on the nine topics/categories (as found in the first part of the study) were analyzed, using the PubMed database as the primary tool. The keywords were specific to each category used in the search with the Boolean “AND” to link them to COVID-19 and India, e.g., “Migrant workers” AND “COVID-19” AND “INDIA; “Announcement of the Lockdown” AND “COVID-19” AND “INDIA”; “Panic Buying” AND “COVID-19” AND “INDIA”; “Stressor and Coping Strategies” AND “COVID-19” AND “INDIA”; “Mental Health” AND “COVID-19” AND “INDIA”; “Frontline workers” AND “COVID-19” AND “INDIA”; “Stigma and Harassment” AND “COVID-19” AND “INDIA”; “Crime during lockdown” AND “COVID-19” AND “INDIA”; “Religious Congregation” AND “COVID-19” AND “INDIA”; “Government of India” AND “COVID-19” AND “INDIA”. The search strategy yielded 468 articles in total, which were used as possible sources of analysis, after which inclusion and exclusion criteria were established. 

#### 2.3.1. Inclusion Criteria

1. Empirical research on the relationship between the nine categories of the topic.
2. Opinions, reviews, and original research papers published from 2020 to the present, and with a study population specificity of Indian people.
3. Papers addressing the respective category during COVID-19 in the Indian context.

#### 2.3.2. Exclusion Criteria

1. Papers addressing the issues related to COVID-19 and India in other contexts than those of the nine categories.
2. Papers were excluded when published in the context of any other country than India.

### 2.4. Flow Chart

The PRISMA Guidelines were followed for assessing and reporting the current study [22]. Our initial search identified 468 articles from the PubMed database. During the screening process, 27 duplicate articles were removed. Of the remaining 441 articles, 358 articles were discarded after reading the abstract, based on inclusion and exclusion criteria. Reports assessed for eligibility were N = 83 articles, which were included in the review, and these were categorized into the different categories related to COVID-19 in the context of India (see Results section, Table 2). 

### 2.5. Statistics

GraphPad software was used to analyze differences between the frequency of two categories by employing the chi-squared or Fisher’s exact test. In all cases, *p* < 0.05 was considered statistically significant.

## 3. Results

### 3.1. Newspaper Content Analysis

The newspaper coverage analysis was based on 129 reports related to COVID-19 published two weeks after the announcement of the lockdown and used in the current analysis. The reports were read multiple times to reveal patterns in the content and based on the nine categories identified (Table 1 and Table S1). In each category, a few related news/stories are here presented. Table 2 presents the categories according to the number of reports. It was evident that the maximum number of news articles published were related to stress and coping strategies for middle/upper class individuals (*n* = 39), followed by problems of daily wagers/migrant workers (*n* = 24), the Nizamuddin outbreak (Religious congregation) (*n* = 17), the role of authorities (*n* = 14), and the effect of mental health (*n* = 11). In the following paragraphs, each one of these categories is presented and discussed. 

### 3.2. Announcement of Lockdown and Panic Buying

The announcement of the sudden lockdown and how people responded to it was covered by seven newspaper reports. The main topics included were: (a) *panic buying*, (b) *overpricing customers*, and (c) *anxieties over survival and uncertainty.* There was evidence that people engaged in undesirable behavior during the COVID-19 pandemic, such as hoarding essential items, food products, sanitizing/disinfecting products, and hoarding medicines [105,106]. There were also reports that many shopkeepers were selling essentials at an inflated price [107]. Many families were concerned about their survival without having enough stock at home for 21 days due to movement restrictions. In one article, members of the public also said that they should have given us some time before announcing the lockdown. This anxiety and fear resulted in panic buying and may have occurred because of the role played by the media and not receiving enough assurance from the authorities [106].

At the scientific level, there were few studies (only four) published that understood the panic buying behaviors among individuals during epidemics or pandemics. A perception of scarcity, a feeling of insecurity and instability, losing control over the environment, uncertainty, safety-seeking behavior, government action, rumors, and misinformation were the most common factors linked with panic buying. However, there is a dearth of research on panic buying and preventive behaviors during epidemics and pandemics, especially in the Indian population.

### 3.3. Daily Wagers and Migrant Laborers

The troubling picture that received enough public attention during the early days of the lockdown was the plight of migrant laborers moving back to their native villages. During the two weeks following the lockdown, 23 press reports described the dramatic scenario, with titles for these stories such as “Humanitarian crises” and “Wave of tragedies”. There were two subcategories, referred to as *“transportation and basic survival*”, and “*health-related issues*” under this category. 

#### 3.3.1. Transportation and Basic Survival

The sudden enforcement of the lockdown further disadvantaged already vulnerable populations of the society. Daily wage workers became jobless and struggled to survive [108], as they were largely employed in the informal sectors without any kind of protection under the Indian labor laws [109]. With no income for the past few days and no hope for the future, thousands of people started walking back to their native villages. 

In a report, a 30-year-old man who was walking with his 11-year-old daughter stated,

*“People like me need daily earnings to survive. Those who take decisions should have thought about us before shutting down public transport overnight.”*

Another news featured how “70 people huddled in a water tank to move out of the cities” and “a pregnant woman had to walk up to 200 km to reach her home”. There were many heart-wrenching stories such as these all over India. The only concern for most of the migrants reported was to reach their homes in any manner, as hunger, thirst, and a sense of being destitute were common apprehensions after the announcement of lockdown. In India, 120 million people feared to lose their jobs or economic activities, and many of them were highly vulnerable [10]. 

The plight of the internally displaced persons is comprehended verbatim from the news report—“*If we are destined to die, we will die among our people…in our villages.*”

#### 3.3.2. Health-Related Concerns

Due to the sudden announcement of the lockdown, thousands of migrant workers started assembling near the bus/train station in the major cities of India without any mandatory social distancing. Implementation of public health measures becomes difficult in congested places, and adequate hygiene and sanitation were not possible to maintain [108]. In another report, migrants who were returning to their homes were sanitized with bleaching agents (diluted mixture of sodium hydrochloride and water) and soon after, children complained of itching in their eyes, and some women developed rashes. The feeling of fear, helplessness, hopelessness, and uncertainty lead to increased stress and other psychological issues among many people [110,111]. Maintaining social distancing was another category that was discussed in many of the newspaper stories. 

In India, 5.51% of the total population live in slum areas [112] that are overcrowded and lack basic amenities such as clean water and toilets.

*“We know about quarantine and social isolation, but these are luxuries the poor cannot afford, we live together in one small, rented room”*—reported one of the news stories based on interviews of some of the residents of a poor neighborhood.

To put it bluntly, it was a lethal combination of hunger, poor sanitation, agony, trauma (physical and psychological), and desperation to return home [113], to live with dignity and to potentially seek death with dignity. Some other groups, such as sex workers, became marginalized further, as reported by three of the news stories, because of the double jeopardy of being poor and belonging to a stigmatized group.

The findings from the scientific literature were very similar to newspaper articles published during the time of the lockdown. The sudden industrial closure and informal labor arrangement pushed many migrants/internally displaced persons to travel back to their city of origin without transportation, food, or medications, and with no financial assistance to fulfill their basic necessities [37]. Internal migrant workers usually come from groups recognized as low-income individuals or disadvantaged populations, coming from rural geographic areas to work in urban areas on a temporary seasonal basis. According to Choudhari (2020), there are certain predispositions of IMWs/IDPs that affect their mental health, such as susceptibility to new communicable diseases, pre-existing occupational morbidities as a risk factor, limitation of use quarantine and social-distancing rule, vulnerable to develop mental disorders, pre-existing mental health issues, economic constraints due to work-loss, and absence of effective laws for unorganized sector workers [30].

Migrants have experienced multiple losses, including loss of jobs, limited food for the family, no work/resources in their villages, and sudden loss of income. The pandemic not only affected their occupational life, but also impacted their relationships with their family and friends [28]. Migrants were also blamed, stigmatized, and discriminated against for spreading the COVID-19 virus, and were targeted, even after completing the 14 days of quarantine [34]. The COVID-19 pandemic and the mass exodus caused psychological distress, severe anxiety, and depressive symptoms in migrant workers, and it inversely affected their mental health [41]. It is crucial to have extensive, structured, and comprehensive welfare policies for public and mental health for all strata and hierarchies of Indian sectors so that the vulnerable population will not be further marginalized.

### 3.4. Stressors and Coping Strategies for Middle/Upper Class Individuals

There were 39 reports concerning the everyday stressors and coping strategies practiced by advantaged groups to deal with lockdown/quarantine blues. There were two major subcategories under this category. (a) *Stressed because of lockdown*: Many people felt that they could not go for a walk, or to the gym, or practice others forms of exercise, could not be able to attend festivities, and some were missing their office bonding as well. Some people were stressed about their health and physique while others reported the pressure of staying at home is real and called it “quarantine blues”. During the lockdown, outdoor activities such as educational institutions, offices, recreational centers, clubs, and sports were totally banned. Perhaps a decline in active participation in pleasurable outdoor activities adversely affected well-being [114], leading to distress. It has also been surmised that the pressure of social distancing increased the risk of loneliness, isolation, nightmares, and anxiety [115]. 

*Reinventing oneself and coping strategies*: For many, the lockdown provided a chance to become closer to their partners and other family members. Furthermore, most of these people are working from home and feel more productive than ever as working from home feels like “*being a boss*.” For many, lockdown is also a chance to enjoy one’s existence, the ability to cherish bonds with others, engage in long-forgotten hobbies, neglected passions, and unfulfilled dreams [116]. Interestingly, digital portals were all set to deal with the lockdown. Most of the materials have been made available online at this time be it online classes or virtual workout sessions to online pubs and concerts for enjoyment at home. Individuals spent their time in indoor activities such as listening to music, reading books, and watching Netflix/Amazon prime. Similarly, Zhang reported in his study that most participants in China mentioned that they were paying more attention to their mental health, spending more time relaxing, resting, and exercising after the onset of the pandemic [117]. However, this situation is far from normal, and many professionals are providing suggestions on how to stay calm and healthy at home during a pandemic [118]. There were news reports related to coping strategies for dealing with social distancing as well as quarantine blues.

There are many studies conducted on stress during the pandemic but very few emphasized problems/stressors and coping strategies used by individuals living in urban areas and belonging to the middle and upper strata of society. The most common problems and stressors faced by individuals were routine disruptions, uncertainty about the future, fake news, and misinformation, worry of shortage of medical supplies and groceries, loss of productivity, health concerns for themselves and loved ones, emotional problems, and social distancing [42,43]. Several coping strategies were used to deal with the pandemic stressors, which is very similar to the findings of the newspaper report. The most common coping strategies were using self-health monitoring, telephonic checkups with the physician, indoor physical exercises, and going outside. Distraction has also been found to be very effective, including watching TV, cooking food, reading, and listening to music. On the other hand, spiritual practices and social support were found to be protective factors during the time of the pandemic.

### 3.5. Effects on Mental Health

Mental health issues turned out to be one of the highlighted topics in the newspapers since the announcement of the national lockdown. There were three subcategories identified out of 11 news reports, including: (a) *General anxiety related to COVID-19 virus*, as people have not experienced anything of this kind and magnitude in their lifetime, this has resulted in severe uncertainty, fear [104], and anxiety [111]; (b) *Isolation and quarantine*, were the other subcategories reported by the newspaper; (c) *Lockdown and the pre-existing mental health issues*, were the next subcategories under this category.

According to the news pieces published during the review period, the pandemic and the lockdown had a more serious effect on people who have pre-existing mental illnesses, especially obsessive–compulsive disorder, depression, anxiety disorder, and insomnia [119]. There has been a significant increase in substance-use-related withdrawal symptoms among individuals, changes in sleeping and eating patterns, and deteriorating conditions of mental health, which may result in suicide [120]. Children’s mental health worsened due to COVID-19 anxiety and fear at the same time, as parents were finding it hard to meaningfully engage the children during the lockdown. During the lockdown, marriages have been under severe strain, and there was pressure on couples to sustain their relationships [121].

### 3.6. Safety of Frontline Healthcare Workers

In this topic, 11 reports focused on the shortage of safety kits leading to fear amongst healthcare workers. There was a general shortage of personal protective equipment (PPE) in the entire country during the initial days of the lockdown. Many doctors have been infected by COVID-19 due to the lack of masks, gloves, and PPE. 

The anger was seen in many frontline healthcare workers, as one of the doctors said “*Do you send soldiers to war without guns? Then why are you sending doctors to fight this war without proper kits*.” 

Another doctor quipped “*healthcare providers should be rather asked to sign their death certificates before going on this suicide mission… we’re ready to serve but not without protection*!” 

Four doctors from a prestigious hospital in Delhi resigned due to their failed demands for PPE. Healthcare workers are at significant risk of contracting illness during the conduct of their professional duties because of their proximity to individuals infected with the pandemic disease. It was found that healthcare frontline warriors reported significantly heightened fear of COVID-19 as compared to other groups of respondents [122], and approximately 400 Indian doctors have lost their lives while treating individuals with COVID-19 [123].

Most of the scientific literature discussed the mental health issues faced by frontline healthcare workers. Healthcare workers could not afford the luxury to be with their families or in quarantine [124]. Most of the literature highlighted the shortages of essential hospital equipment that make them more vulnerable to contracting infections. Doctors and nurses, not only experienced poor psychological well-being, but were also stigmatized and discriminated against [90]. 

### 3.7. Crime during Lockdown

The COVID-19 pandemic has impacted crime levels covered by three press reports during the lockdown. There were two kinds of categories under this: (a) *drastic fall in crime*, as most crimes reduced by half and some even reduced by 80%, especially street crimes, robbery, molestation of women, and road traffic accidents; (b) *lockdown became terrible for some women* who were staying with abusive partners. According to one of the reports, 13 women have registered complaints of rape and attempt to rape, 69 women sought protection from violence, and 77 others reported various complaints related to dowry and harassment. Ironically, crimes on the roads have remarkably reduced but the home is no longer safe for so many women. There is convincing evidence that domestic violence/intimate partner violence has serious consequences for mental health including PTSD [125].

In the crime section, the scientific studies found a significant increase in daily crimes at home, including domestic violence. The literature highlighted the significant increase in spousal violence, and abuse in form of physical, psychological, or/and sexual. In a recent report by UN women, the COVID-19 pandemic led to increased violence against women. Lockdown measures and the lack of social services have left many women alone with their violent spouses at home [126]. Out of four women, at least one woman reported a form of domestic violence during the phase of the lockdown in India. 

### 3.8. Religious Congregation Amid the COVID-19 Pandemic

An infamous religious congregation, known as the Nizamuddin outbreak, became a COVID-19 hotspot during the first phase of the national lockdown. There were 17 reports published on the print media for this religious congregation; two kinds of news were reported under this category: (a) *negligence on the part of organizers/participants* and (b) *conspiracy theories*. There was a religious conference in the month of March, which was attended by many people from India and abroad. The reports stated that almost 1500 participants stayed in the mosque even after the conference was over. More than 2000 people were later evacuated from the mosque and sent to the hospitals and quarantine centers. Some people felt that one particular religious group conspired to bring Coronavirus to India, and some people even called this group a Corona Jihad. Conspiracy theories help people to explain big events with proportionally large causes, and people are more likely to believe in conspiracy theories about events with serious consequences [127]. Conspiracy theories may also fuel hostility towards the group seen as responsible for bringing the virus to India, and there were some reports that members of this community faced discrimination and some form of violence.

From the scientific literature, there were only two studies published on religious congregations that happened in India in the month of March 2020. These articles also emphasized the conspiracy theories after the large gathering of the religious congregation (*Tablighi Jamaat*) of the Muslim community in India led to anti-Islamic sentiments. The Islamophobic sentiments against the community were reported, including the denial of Muslim patients in hospitals, separating patients based on religion, and boycotting the business of Muslim community members [83]. Discrimination, xenophobia, and prejudice towards the Muslim community were found to be significant, which is associated with poor well-being among the Indian population [84]. The role of the media was also highlighted to control misinformation, hate speech, and fake news, and influencing prejudice and stigma during the pandemic. 

### 3.9. Harassment and Stigma

Many forms of stigma and discrimination have been witnessed since the identification of the pandemic. Four press reports highlighted the presence of stigma and harassment in the general public. Some of these stories were related to doctors and nurses serving patients who tested positive for COVID-19 and experiencing harassment from landlords because of fear. Frontline healthcare workers faced stigma and unfortunate experiences such as avoidance by the community due to the stigma or fear of contracting the virus [128]. There were some stories on the fear of contracting the virus that has been instrumental in giving rise to stigma and xenophobia [9] directed toward those whom others might suspect as carrying the virus. There were a few incidents where northeast Indians have been subject to prejudice and discrimination because of their mongoloid features of East Asians [129]. They were also called “Coronavirus” or even “Chinese”. There is evidence that greater fear and perceived threat are associated with greater intolerance and punitive attitudes toward out-groups [127]. 

### 3.10. Role of Authorities

A few articles were related to the help being provided by the authorities to the public in general as well as to daily wage laborers. A total number of 14 articles were published on the role of various authorities during the current pandemic. The center and state governments of India tried to ensure stable supplies soon after the lockdown was announced. Many initiatives, such as a corona package worth Rs. 1.7 Lakh crores (USD 24 billion 476 million 320 thousand) announced by the finance minister of India. Free food grains for the poor and free meals for 1 lakh beggars (0.1 million) were also announced by the governments. Despite all these announcements to help vulnerable sections of societies, the ground situation remained very grim. Some of the news items also focused on the positive role played by the police during the period under review as “*the protectors turned providers.*”

There are only two scientific articles found on the role of government and authorities in dealing with the COVID-19 pandemic. Immediately after the Lockdown, the government announced a public transfer policy Pradhan Mantri Garib Kalyan Yojana (PMGKY) which benefitted farmers, women, individuals with low income, and economically disadvantaged populations [103]. However, the study by Abbas (2021) suggested that despite implementing strict policies in India, the government could not be able to deal with the brutality of COVID-19 [104]. 

## 4. Discussion

The COVID-19 pandemic and the restrictions imposed thereafter had a burgeoning impact and led to a major disruption in all aspects of life [130]. To curtail the spread of the infection, many countries imposed a nationwide and global lockdown soon after the declaration of the pandemic by WHO [131]. The impact of lockdown has been witnessed globally regarding the economy, physical and mental health, employment, food security, tourism and hospitality, and the environment [132]. Despite the pandemic and the lockdown having global effects, the impact was worse in developing countries [133]. 

From the newspaper coverage analysis, it was observed that there was a sense of panic and uncertainty among the general public after the announcement of the sudden lockdown. Preparedness and contingency plans are important to mitigate the spread of infections. Containment strategies such as lockdown, curfew, and shutdown are important, and need to be imposed by the authorities after analyzing the scenarios. Alternative adaptive ways should be employed for carrying out essential activities [134].

Similar to other developing and densely populated countries, India is a multi-stratum society, and the effects of the pandemic were seen differently for each stratum. Individuals with low income or economically marginalized are more susceptible to developing COVID-19 and faced the wrath of the pandemic. The distress is not limited to the worry of contracting an infection or movement restriction but to the survival and fulfilling necessities. 

Daily wage workers and migrants have been affected the most due to the sudden lockdown due to casual day labor and informal employment [135]. Many low- and middle-income individuals will lose their jobs and economic support due to the COVID-19 outbreak that also have been seen in previous outbreaks [136]. Gumber and colleagues [137] highlighted that poor and underprivileged individual are more vulnerable and suffer the most from any infectious diseases. On the other hand, we observed the middle- and high-income groups of the society were more concerned about routine disruptions, scheduling the day, duration of lockdown, fear of infections, feelings of frustration and boredom, inadequate supplies, and inadequate information [138].

The other vulnerable groups identified in the present study are women, children, and ethnic and religious minorities in the country. They are more susceptible to experiencing abuse, violence, discrimination, prejudice, and mental health issues. The United Nations Population Fund (UNFFA) estimated a record of approximately 31 million additional cases of gender-based violence in six months of lockdown [139]. Violence against women is the most prevalent violation of human rights and is a global public health concern. According to WHO (2021), on average, the violence against women prevalence estimated at 852 million women who were aged 15 years and older (almost one in three women) and witnessed intimate partner violence and non-partner sexual violence or both. The lifetime prevalence has been highest estimated in least-developed regions, such as Oceania, sub-Saharan Africa, and South Asia [140]. 

Violence against women (VAW) is preventable. There is an urgent need to implement preventive actions and policies including multilevel strategies. UN Women and WHO developed the RESPECT women framework (2019) using seven strategies to prevent VAW- it includes R for Relationship skills strengthened; E for Empowerment of Women (economic, behavioral, and social skills building); S for services ensuring including legal, medical, police, and social services for survivors; P for poverty reduction; E for environments made safe in schools, public places, and work environments; C for child and adolescent abuse; and T for transformed belief, attitudes, and norms [141]. In the issue of Lancet Public Health, Alexa Yakubovich and colleagues highlighted the importance of housing interventions in terms of mental health outcomes, perceived safety, and dealing with partner-related stress in women experiencing IPV. However, Rachel Jewkes emphasized the need for systematic evaluations of housing interventions to be conducted in low- and middle-income countries [126]. 

Infectious diseases have been linked to stigmatization and marginalization throughout history. Discrimination against ethnic and religious minorities has risen as a result of the COVID-19 pandemic. This includes microaggression and violence to systemic patterns, such as the exclusion or debarring of individuals in society. Discrimination and scapegoating against the traditionally targeted populations should be eliminated and policies should be framed based on equality and justice [142]. 

Mental health issues were found to be prevalent in general, especially among healthcare workers, and the marginalized groups. The most common mental disorders reported were depression, anxiety disorders, sleep disorders, and post-traumatic stress disorder [143]. A study by Rajkumar (2020) highlighted the need for mental health services, especially for the vulnerable population to deal with the adverse psychological effect of epidemics and pandemics. Mental health and psychological well-being have been severely disturbed by the pandemic and the lockdown [119]. Mental health actions need to mitigate the psychological consequences of the pandemic including the adoption of a “Whole-of-Society” approach in the COVID-19 national response to promote, protect, and care for mental health, provide emergency psychosocial support, and to enhances coping skills of the public for recovery and rebuilding of communities [144].

The media played a significant role from the very beginning of the pandemic in disseminating information about the prevention of the infection and opinions about the pandemic to the general public. Social media helps as contagion and vector, for surveillance and monitoring, and disease control [145]. There have been several studies conducted on social media content analysis for various platforms such as Twitter, YouTube, and Instagram [146,147,148,149]. With the help of newspapers, the information not only reached the urban cities but also the rural areas or communities. Local newspaper coverage become the most substantial source for the public. The media identified the deepening crisis during the lockdown phase and put the plight of the most vulnerable forefront. In this respect, *The Times of India* Newspaper has been considered for the media coverage analysis. 

However, there is no denying many social media platforms the news originated from users and endorsed fake information and conspiracy theories that led to adverse effects on people’s mental and physical health. Moreover, conspiracy theories and beliefs have been linked to adverse mental health issues [150,151].

This study was carried out by taking newspaper reports from one newspaper and only for a specific duration. Additionally, the possibility of retraction of the news/media reports and scientific literature are common. However, the purpose of the present study was only to understand the role of newspaper in identifying the issues reported due to the sudden lockdown. These factors could be considered a limitation of the current study. An extensive set of reports and other relevant media could also be used to follow up on the news to study the long-term impact of the lockdown in India and establish the reliability of the news reports. 

## 5. Conclusions

The COVID-19 pandemic has caused severe socioeconomic and mental health issues all over the world. All social classes have been impacted, but lower-income people have been particularly vulnerable. The COVID-19 outbreak has endangered people in impoverished countries since the bulk of people depends on their daily income for their ability to survive. The other vulnerable groups found in the study were children, women gender, migrants and daily wage earners, ethnic and religious minorities, and individuals from disadvantaged societies. These groups are more vulnerable to contamination with COVID-19 infection, developing mental illness, and more likely to face discrimination and stigma. Such pandemics and public health emergencies affect humans in general but make the vulnerable population more marginalized. 

The newspaper coverage analysis provided evidence of the uneven implications of lockdown among different stratum of society. Extended duration of lockdown, disruption of routine, boredom, and absence of social life were the major issues associated with serious mental health complications in the middle and upper-income class. On the contrary, unemployment, no financial security, poor healthcare services, harassment, and stigmatization are associated with mental health outcomes in internally displaced individuals. 

Importantly, the gap has been identified between the newspaper coverage analysis and the scientific literature data. Most of the original articles published in the past two years focused on the general population, but very few on the marginalized sections of society. The newspaper coverage reflected the contemporary issues faced by the Indian population at the time of the lockdown, especially the marginalization sections, and provided information about all sections and fabrics of the society. It is indeed crucial to employ that knowledge of individual as well as social scenarios in framing policies and strategies. Therefore, it is essential to prepare and adopt policies that would fit the society as a whole and provide protection to all the stratum of the society. For effective implementation of the strategies and policies designed by the governments need to be provided with appropriate assistance. 

## Figures and Tables

**Table 1:** Categories and subcategories are identified in the content analysis.

**Categories and Subcategories** **(Subcategories Are in Parentheses)** **• Relevant News/Reports (See Supplementary Table S1)**
**1. Announcement of Lockdown** (Panic buying, overcharging the customers, and general anxiety regarding how to survive without any prior planning)
**2. Daily wagers and migrant workers** (Transportation and basic survival and health-related concerns)
**3. Stressors and coping for middle- or upper-class people** (Stressed because of lockdown and coping and reinventing oneself)
**4. Effect on mental health** (General anxiety related to COVID-19, isolation, quarantine, lockdown, and pre-existing mental health issues)
**5. Safety of medical frontline workers** (Shortage of safety kits and fear of becoming infected)
**6. Crime during lockdown** (Falling crime rates on the road and domestic violence rising)
**7. Religious congregation** (Negligence on the part of some people and the organizers of the conference, conspiracy theories)
**8. Harassment and stigma** (Harassment and stigma of frontline doctors and nurses, stigma towards others who are suspected of being a COVID-19 patient, prejudice and discrimination towards people of northeastern India)
**9. Role of authorities** (Being helpful, many initiatives by the Govt. agencies, yet the ground reality is not good)

**Table 2:** List of categories according to their representation in the social media reports and scientific outputs (2020–2022).

Categories	News Number (%)	Scientific Outputs (%)		Scientific References	
1. Announcement of lockdown	7 (5.4)	***#	4 (4.8)	***##	[23,24,25,26]
2. Daily wagers and migrant Laborers/IDPs	**23 (17.8)**	*	15 (18.1)		[27,28,29,30,31,32,33,34,35,36,37,38,39,40,41]
3. Stress and coping for middle/upper class individuals	**39 (30.2)**		4 (4.8)	***##	[42,43,44,45]
4. Effect on mental health	11(8.5)	***#	**22 (26.5)**		[46,47,48,49,50,51,52,53,54,55,56,57,58,59,60,61,62,63,64,65,66]
5. Safety of health frontline warriors	11(8.5)	***#	12 (14.5)		[67,68,69,70,71,72,73,74,75,76,77,78]
6. Crime during lockdown	3 (2.3)	***###	4 (4.8)	***###	[79,80,81,82]
7. Religious congregation	17 (13.2)	***	2 (2.4)	***###	[83,84]
8. Harassment and stigma linked to COVID-19	4 (3.1)	***###	**18 (21.7** **)**		[85,86,87,88,89,90,91,92,93,94,95,96,97,98,99,100,101,102]
9. Role of authorities	14 (10.9)	***	2 (2.4)	***###	[103,104]
Total	129 (100)		83 (100)		[23,24,25,26,27,28,29,30,31,32,33,34,35,36,37,38,39,40,41,42,43,44,45,46,47,48,49,50,51,52,53,54,55,56,57,58,59,60,61,62,63,64,65,66,67,68,69,70,71,72,73,74,75,76,77,78,79,80,81,82,83,84,85,86,87,88,89,90,91,92,93,94,95,96,97,98,99,100,101,102,103,104]

In bold, the two main categories. Statistics: chi-squared or Fisher’s exact test, * *p* < 0.05 *** *p* < 0.001 vs. 1st main category; # *p* < 0.05, ## *p* < 0.01, ### *p* < 0.001 vs. 2nd main category.

## Data Availability

Not applicable.

## Supplementary Materials

The following supporting information can be downloaded at: https://www.mdpi.com/article/10.3390/bs12100348/s1. Table S1. Categories and sub-categories identified in the content analysis of media according to the timeline.
