# Supplementary material for: Uneven Implications of Lockdown Amid COVID-19 in India: From Harassment, Stigma, Crime, and Internally Displaced People to Stress and Coping Strategies in the Middle/Upper Class
Source: Behav Sci (Basel). 2022 Sep 21;12(10):348. doi: 10.3390/bs12100348 (PMC9599041; doi:10.3390/bs12100348)
Supplement: Supplementary file 1 [file behavsci-12-00348-s001.zip › behavsci-1879662-SI.pdf]

behavioral sciences

MDPI

Supplementary Table S1. Categories and sub-categories identified in the content analysis of media according to the timeline

Categories and sub-categories
(Sub-categories are in the parenthesis)
- Relevant news/reports

1. Announcement of Lockdown and Panic Buying
(Panic buying, overcharging the customers and general anxiety to survive without any prior planning)
- People in cities anxious for necessities outside general store/departmental store
- People scramble for essentials after the Prime Minister's speech
- Essential services will be there
- Chaotic Monday (First day of the Lockdown)
- There is hardly anything left in most stores
- Panic buying
- Stocking
- Selling essentials at an inflated price
- The poor are not able to pay for pulse and rice

2. Daily wagers and Migrants workers
(Transportation and basic survival and Health related concerns)
- Suddenly jobless, migrant workers on a long walk home
- 70 people huddle up in a container to move out of a city
- With no transport, migrants make the long march home and walk hundreds of kilometers
- No vehicles to go out from the cities due to sudden lockdown
- 200 used boats to reach home during lockdown
- A pregnant woman walks 200km to reach home
- Man cuts off the plaster, still has 240 km to go, began hobbling his way home
- Humanitarian crises
- Joblessness due to lockdown
- Little or no money or food
- Next pay would come? Not so sure
- No income, earnings have dried up because of lockdown
- Food/Shelter/Losing jobs and No means to feed their families
- Health crisis
- Homeless
- Destitute
- Fighting for survival
- Hunger
- Fear
- Tiredness
- Helplessness
- Uncertainty
- "What will you eat, if don't earn"
- Out of job, food, and luck
- Sex workers run out of food, and essentials, and face stigma as well
- Social distancing at slum: A cruel Joke
- They should see the number of people living in each house and distribute food accordingly (A possible way out)
- Lives in camp or isolation
- Massive evacuation as the huge mass of migrants heads out of it

behavioral sciences

MDPI

- Migrants sanitized with bleach agent, sprayed with sodium hydrochloride
- Schools become a temporary home for migrants
- Future is too fancy a dream right now

3. Stressors and coping for the middle or upper class of people
(Stressed because of Lockdown and Coping and reinventing oneself)

- Travel has stopped not creativity
- Music
- Playlists
- Playing together
- Digital events
- Online Pubs
- Tik Tok raves
- Exercising together
- Fitness enthusiasts turn to virtual workout sessions
- The global spike in gaming
- Learning guitar
- Online classes
- Netflix series
- Watching and reading about the end of the world apocalyptic stories
- Time to organize old photos
- Zoom video call
- Quarantine baking
- Housekeeping activities, like cleaning and cooking
- Chopping tasks and washing the dishes are meditative for some
- Employing humor as a balm
- My half-full glass attitude comforts me
- Exercise
- Humming
- Gardening becoming therapeutic
- Indoor games
- Staying home is a religious duty
- Staying home best worship at the times
- Start mental health counseling via phone or internet
- Super rich buying hotels to self-isolate
- Getting closer to family than ever before
- Bonding time with our husband, we will also be doing cleaning together
- We may become more productive working from home

Work from home like "being in big boss"

4. Effects on mental health
(General anxiety related to COVID-19, Isolation, Quarantine, Lockdown, and pre-existing mental health issues)

- Pandemic also takes a toll on mental health
- Anxiety, depression & fear are some common symptoms
- Fear and worry about your health and your loved ones.
- Changes in sleep or eating patterns
- Worsening of chronic health problems
- OCD, Depression
- Children's mental health getting contracted stomachaches." If someone sneezed on me, I thought I was going to get it."
- Challenge to keep restless teenagers gainfully occupied
- You are bound to quarrel with your spouse
- Will the marital relationship survive the lockdown?

behavioral sciences

MDPI

- From hallucinations to palpitations, withdrawal symptoms
- Corona anxiety
- 22-year youth committed suicide
- A man committed suicide after an argument with the wife
- A man committed suicide by jumping in front of a train two days ago after the residents of his area suspected him to have symptoms of Covid 19.

5. Safety of Medical frontline workers

(Shortage of safety kits and fear of getting infected)

- 4 Hindu Rao (A Hospital in Delhi) doctors resigned after their demand was not met for PPE
- PPEs in short supply, and medics get infected
- Frontline medics fume over protective gear
- Doctors in war without their armor
- Compared to previous weeks, the supply of PPEs has been better, however, it is still a matter of concern.
- Docs, and nurses at NDMC hospital (Based in New Delhi) quarantined
- A Delhi hospital was sealed after 18 staff tested positive
- Lack of PPE, Medics avoid loos, drinking water for 6-7 hours

6. Crime during Lockdown

(Falling crime rates on the road and domestic violence on rising)

- Crime rates dip 80% as People stay in
- Domestic violence/abuse up substantially
- Extramarital affairs rise amid lockdown; extramarital app claims subscriptions are up by 70% in India
- Hackers are using this crisis to gain access to sensitive and personal information

7. Religious Congregation

(Negligence on the part of some people and the organizers of the conference, Conspiracy theories)

- *Sudden outbreak and detection of multiple coronavirus cases at Nizamuddin (New Delhi)*
- Disaster at Nizamuddin (New Delhi)
- Over 500 vicinities under Nizamuddin surveillance
- FIR against the man who led the congregation
- Fear they may create new clusters
- 398 patients linked to religious congregations tested positive and the number rising
- 2361 people evacuated out of the mosque
- Over 1000 members of the religious congregation infected, account for 30% of all Indian cases

8. Harassment and Stigma

(Harassment and stigma of frontline doctors and nurses, stigma towards others who are suspected of COVID-19 patient, Prejudice and discrimination towards people of north-eastern Indians)

- Health workers face social stigma
- Patients asked me (nurse) to be replaced, maybe I too have Corona.
- People shame us by calling us corona (Some girls from the North-East part of India)
- Harassed and abused by locals
- Stop racism

9. Role of Authorities

(Being helpful, many initiatives by the Govt. agencies yet the ground reality is not good)

- Centre and state governments seek to ensure stable supplies
- Free food grains for the poor in Rs. 1.7L crore corona package
- Govt. plans meals for 1 lakh beggars
- Police step in to feed those left jobless due to lockdown
- Give shelter to stranded migrants and make them stay put
- When protectors turn providers
- Don’t leave, we will provide food

behavioral sciences

MDPI

- Governments blamed each other
- Seal borders to stem the migrant exodus, ensure shelter, food, wages, rent relief, and central government to state governments
- A meal cooked for lakhs of people
- Officials let things out of control
- The ground situation is not showing what the government is saying
